# Horner’s Syndrome after Superficial Cervical Plexus Block

**DOI:** 10.5811/westjem.2015.2.25336

**Published:** 2015-04-06

**Authors:** Stefan Flores, Christine Riguzzi, Andrew A. Herring, Arun Nagdev

**Affiliations:** * Highland Hospital-Alameda Health System, Department of Emergency Medicine, Oakland, California; † University of California, San Francisco, Department of Emergency Medicine, San Francisco, California; ‡ Alta Bates Medical Center, Department of Emergency Medicine, Oakland, California

## Abstract

Ultrasound-guided nerve blocks are becoming more essential for the management of acute pain in the emergency department (ED). With increased block frequency comes unexpected complications that require prompt recognition and treatment. The superficial cervical plexus block (SCPB) has been recently described as a method for ED management of clavicle fracture pain. Horner’s syndrome (HS) is a rare and self-limiting complication of regional anesthesia in neck region such as brachial and cervical plexus blocks. Herein we describe the first reported case of a HS after an ultrasound-guided SCPB performed in the ED and discuss the complex anatomy of the neck that contributes to the occurrence of this complication.

## CASE REPORT

A 20-year-old male presented to the emergency department (ED) with right shoulder pain and deformity after falling from his bicycle. Exam was notable for swelling and tenderness overlying the right clavicle with a comminuted mildly displaced clavicular fracture confirmed by plain radiography. The patient complained of severe pain unrelieved by initial parenteral opioids. For improved pain management, an ultrasound-guided superficial cervical plexus block (SCPB) was performed.^1^,^2^

### Ultrasound-guided SCPB

The patient was placed on continuous cardiac monitoring. Placement of a high frequency linear transducer (13-6 MHz, SonoSite™ M-Turbo, Bothell, WA) was approximated by palpation of the superior pole of the thyroid cartilage (C4 level), and visual approximation of the midpoint of the sternocleidomastoid muscle (SCM), from mastoid to the clavicle.

**Figure 1:**
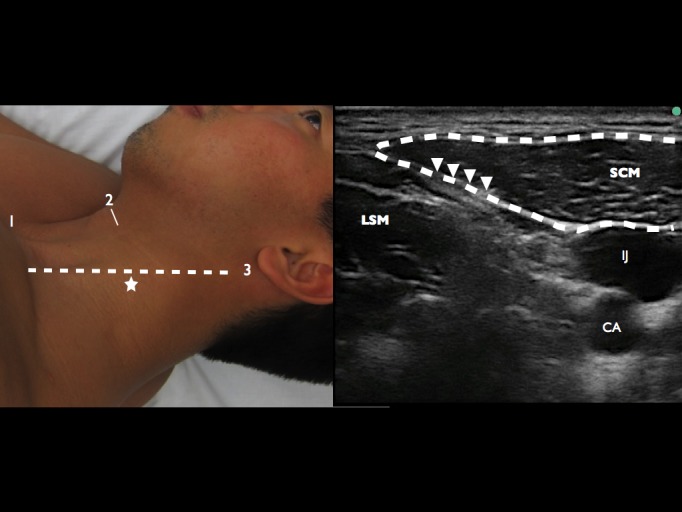
*A*, Key surface landmarks include the (1) sternal notch, (2) superior pole of the thyroid cartilage, (3) the mastoid process, and (dashed line) the posterolateral border of the sternocleidomastoid muscle (SCM). The injection site is marked (star). *B*, Survey ultrasound scan showing the tapering posterolateral border of the sternocleidomastoid (SCM) outlined in red, the internal jugular vein (IJ), the carotid artery (CA), and the levator scapulae muscle (LSM). The superficial cervical plexus just deep to the muscle is marked (arrow heads).

**Figure 2:**
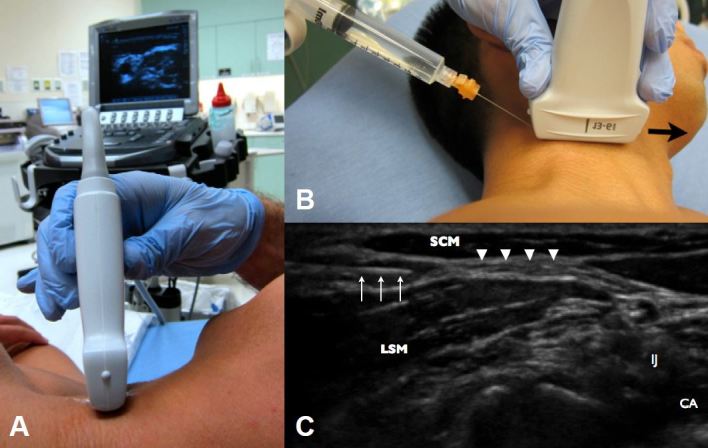
*A*, Probe positioning for the in-plane approach in the lateral decubitus position. *B*, Needle injection and proper orientation of probe marker during superficial cervical plexus block. The arrow delineates the probe marker orientation. *C*, Ultrasound image of needle injection within the superficial cervical plexus. The arrows mark the needle. The sternocleidomastoid muscle (SCM) noted on the top right, the levator scapulae muscle on top left (LSM), the carotid artery (CA) and internal jugular vein (IJ) on the bottom right, and the injection site marked (arrowheads).

The superficial cervical plexus was identified as the hyper echoic fascia posterior to the SCM and superficial to the levator scapula muscle (LSM) (Figure 1). The area was prepped with chlorehexidine and a skin wheal of 1% lidocaine was injected. The patient was placed in left lateral decubitus with the ultrasound system contralateral to the provider (Figure 2). Using a 25g 1.5-inch standard hypodermic needle, with an in-plane posterior approach, 10mL of 0.5% bupivacaine was injected under the SCM in the fascial space between the SCM and LSM. Aspiration and real-time visualization of anechoic anesthetic was done to prevent intravenous injection (Figure 2).

**Figure 3:**
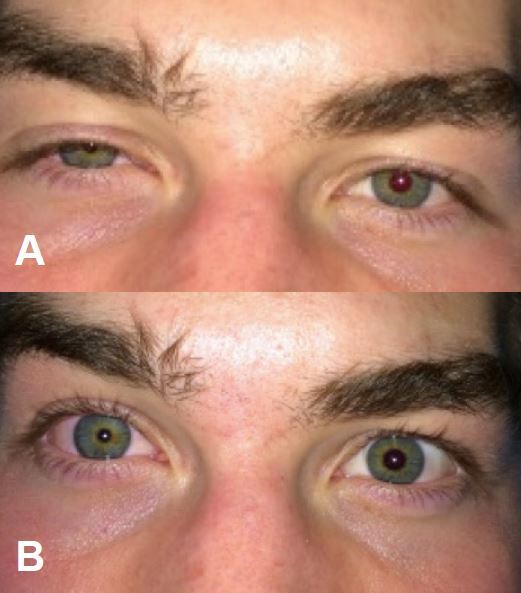
Horner’s syndrome after block placement with *A*, ptosis and *B*, miosis.

Approximately 15 minutes after block placement, the patient had complete pain relief, reporting sensory deficit in the cape region of the shoulder, neck, and skin overlying the clavicle without changes in motor function of the arm. Forty-five minutes later, the patient complained of right-sided facial numbness and was noted to have ptosis, miosis, and conjunctival injection on the ipsilateral side of the block (Figure 3). There was no voice hoarseness, anhidrosis or enophthalmos. The patient was observed in the ED and symptoms resolved 1.5 hours after the block was placed.

## DISCUSSION

Potential complications of a SCPB include Horner’s Syndrome (HS), partial brachial plexus blocks, phrenic and recurrent laryngeal nerve blocks. We believe the HS described here developed as an inadvertent complication of deep spread of local anesthetic after superficial injection, involving the ipsilateral cervical sympathetic chain.^3^ Several studies suggest that the deeper compartments of the neck, (containing the cervical sympathetic chain), and superficial spaces are in communication with each another allowing for potential deep spread of a superficial injection.^4^–^6^

Nash et al.^7^ reported that the investing layer of the deep cervical fascia in the anterior triangle of the neck is nearly non-existent, suggesting that fat and connective tissues surrounding neck neurovascular structures provide direct communication between the subcutaneous tissue and the prevertebral layer beneath the deep cervical fascia. Pandit et al.^4^ further demonstrated that dye injected above the prevertebral layer of the deep cervical fascia penetrates through pores where the nerves pierce the fascia, ending in the deep cervical space. While the precise anatomy has not been completely elucidated, current data supports the concept that the deeper neck compartments potentially communicate directly with the subcutaneous tissue, which explains why in our case the patient experienced a ipsilateral HS after a SCPB. We hypothesize that adhering to three simple precautions can reduce the incidence of HS. More than 5mLs of local anesthetic volume is unnecessary, and placing larger volumes may promote deeper spread of local anesthetic via the anatomic pathways described above. Needle placement at the superior pole of the thyroid (C4 level) should be ensured and not approximated, as was done in this case. Finally, proper depth of injection should be ensured. Needle-tip placement should be maintained just underneath the SCM belly; insertion past the superficial investing fascia may promote the anesthetic to spread into the deep cervical fascial plane potentially involving the phrenic nerve, brachial plexus, and the stellate ganglion.^5^

Finally, it is important to be aware that if HS occurs after the SCPB, providers should consider it self-limiting, requiring only patient reassurance and observation versus urgent neuroimaging to evaluate for an acute stroke.

## CONCLUSION

The SCPB is strategy for ED pain management of clavicle fractures. However, emergency clinicians should be aware that similar complications expected of a deep cervical plexus block could occur with the SCPB, such as HS. To minimize risk of complications of the SCPB, in addition to standard precautions for ultrasound-guided nerve blocks, several precautions should be taken. Clinicians should be aware of the anatomy of the superficial cervical plexus and be familiar with the landmarks, ensuring to stay at the level of C4; the injection should be shallow, just under the SCM belly; and appropriate anesthetic volumes (2–5mL) should be used. The development of HS after SCPB can be frightening to patients and the physician should reassure the patient that it is self-limited and not a sign of intracranial pathology or permanent damage.

## Figures and Tables

**Video:** Needle injection within the superficial cervical plexus.
